# Prognostic Impact of Preoperative Imaging Parameters on Resectability of Hilar Cholangiocarcinoma

**DOI:** 10.1155/2013/657309

**Published:** 2013-06-04

**Authors:** Anthony T. Ruys, Olivier R. Busch, Erik A. Rauws, Dirk J. Gouma, Thomas M. van Gulik

**Affiliations:** Department of Experimental Surgery, Academic Medical Center, University of Amsterdam, Meibergdreef 9, 1105 AZ Amsterdam, The Netherlands

## Abstract

*Objectives*. To evaluate, in hilar cholangiocarcinoma (HCCA), the prognostic impact of specific preoperative radiologic parameters on resectability, metastases, and yield of laparoscopy, and to evaluate the currently used staging systems. *Methods*. Consecutive patients with HCCA presenting in our center from January 2003 through August 2010 were evaluated. Suspicion on lymph node metastasis, portal vein and hepatic artery involvement, lobar atrophy, and proximal extent of ductal invasion was scored. The prognostic value of these parameters for predicting resectability, yield of diagnostic laparoscopy, likelihood of metastatic disease, R0 resection, and survival was assessed. The Bismuth-Corlette classification and MSKCC staging system were evaluated. *Results*. Of all 289 evaluated patients, 158 patients (55%) had unresectable disease based on cross-sectional imaging studies or diagnostic laparoscopy; 131 patients (45%) underwent exploration. 83 patients (64%) underwent resection, of whom 67 (87%) had a radical (R0) resection. Suspicious lymph nodes and involvement of the hepatic artery were important prognostic factors for resectability. Predictive power of the evaluated staging systems was limited. *Conclusions*. Current staging systems predict resectability, but there is room for improvement. Hepatic artery involvement and nodal status might be important factors for prediction of resectability and should be considered in future staging systems.

## 1. Introduction

Hilar cholangiocarcinoma (HCCA), or Klatskin tumor, is a rare cancer arising at the confluence of the right and left hepatic ducts. Radical surgery is still the only curative treatment, although this can only be performed in a minority of patients. Many different imaging techniques are used for staging of HCCA. Nonetheless, only 50–60% of patients who are surgically explored are ultimately amenable to a potentially curative resection, due to peritoneal, nodal, or liver metastases, as well as locally advanced disease [1]. 

Already in 1975 the Bismuth-Corlette (BC) classification was proposed [2], which has been modified in 1992 [3]. This classification system is based on proximal, ductal extent of the tumor. Since the BC classification was not able to predict resectability [4, 5], the Memorial Sloan-Kettering Cancer Center (MSKCC) developed a new presurgical T-staging system in 1998 [6] that also took into account portal vein involvement, lobar atrophy, and ductal extent of the tumor. This staging system was further modified in 2001 [7]. Lastly, in 2009 the TNM staging system for malignant tumors was revised in the seventh edition of the staging manual by the American Joint Committee on Cancer (AJCC) and the International Union Against Cancer (UICC) and also included some changes for extrahepatic cholangiocarcinomas [8]. This system, however, is based on pathological criteria and, thus, is not applicable in the preoperative setting. 

 Over the years, surgical approach, resectability criteria, and options to achieve R0 resection have changed significantly. More specifically, hilar resection in combination with extended liver resection including the caudate lobe is considered essential for long-term survival [9, 10], whereas bilateral ductal involvement (BC type IV) is no longer considered per se a criterion of unresectability. 

Resectability of HCCA patients is defined by factors that are important when considering patients for any major liver resection, such as physical condition, age, and size and function of the future remnant liver. In addition, resectability is defined by factors specific for HCCA, including invasion of the portal vein and hepatic artery, lymph node status, and proximal ingrowth into the segmental bile ducts. These specific factors can be assessed preoperatively with acceptable accuracy [11]. The aim of this study was, firstly, to evaluate the prognostic impact of these specific preoperative parameters on resectability, incidence of metastases, and yield of laparoscopy, and, secondly, to evaluate the currently used staging systems in HCCA.

## 2. Methods

### 2.1. Patients and Staging

All patients suspected of HCCA and managed in the AMC from January 2003 through August 2010 were evaluated. Only patients with a tumor arising at the biliary confluence or the right or left main hepatic ducts were included. Patients with tumors originating in the proximal common hepatic duct were included if the tumor extended into the biliary confluence. As previously described [12], cross-sectional imaging studies such as CT and MRI were used in addition to ultrasound with Duplex to assess liver parenchymal invasion, vascular invasion in portal vein and/or hepatic arteries and hepatic metastases, lymph node metastases, or extrahepatic metastases. Endoscopic ultrasound was not used for staging in this series. After imaging studies were concluded, resectability was discussed in a multidisciplinary, hepatobiliary meeting. Imaging studies were preferably performed in our own center, and imaging studies from referring centers were only accepted, when comparable with our own high-quality imaging studies. 

Patients considered to have potentially resectable tumors underwent further evaluation using staging laparoscopy and, in the last three years of the study, an additional PET-CT. Staging laparoscopy was routinely performed in resectable patients, when feasible, although staging laparoscopy was omitted in some patients with limited BC type I or II tumors, as we described recently in more detail [13]. Since we found no advantage of laparoscopic ultrasound for the staging of lymph nodes and hepatic artery involvement, laparoscopic ultrasound was not used in the current study [14]. Furthermore, ^99m^Tc-mebrofenin scintigraphy [15, 16] was performed in conjunction with CT volumetry to determine function of the future remnant liver. Portal vein embolisation was performed in 6 patients, because the future remnant liver function or volume was deemed insufficient. When no metastases were found during further evaluation, patients were planned for resection, and preoperative biliary drainage was performed, of at least the future remnant liver, either percutaneously or endoscopically.

### 2.2. Laparotomy

During laparotomy, the abdomen was inspected for peritoneal seeding, distant metastases, or lymph node involvement outside the liver hilum, in the hepatoduodenal ligament and along the common hepatic artery until the celiac axis. Suspicious lesions were excised or biopsied and analyzed by frozen section histology. If more causes of unresectability were found, the one discovered first was reported. More specifically, extrahepatic metastases, liver metastases, positive lymph nodes, and locally advanced disease were reported in decreasing order. In case of distant metastasis, only a cholecystectomy was performed. In patients with unresectable disease, biliary drainage was accomplished by definitive internal stenting by either PTC or ERCP using metal expandable stents, usually within the same hospital admission. Chemotherapy was not routinely administered until the results of the ABC-02 trial came out in 2010 [17]. Chemotherapy, palliative radiotherapy, or photodynamic therapy was administered in 38, 12, and 9 patients, respectively. Microscopical confirmation was available in 75% of patients. In addition, most patients without a pathological diagnosis died during followup as described earlier in more detail [18]. 

### 2.3. Assessment of Preoperative Parameters

Because resectability, as defined above, is determined by vascular involvement, ductal involvement, and lymph node status, we primarily evaluated these items for their prognostic impact on resectability. Patients who were unresectable at initial imaging were not included in this analysis, since prediction of resectability in unresectable patients obviously is useless. For every potentially resectable patient we scored the absence or presence of tumor involvement of the portal vein, or hepatic artery, suspicious lymph nodes as well as lobar atrophy, or bilateral ductal involvement. These parameters were retrieved from reports of CT, Duplex ultrasound, and MRCP, in which these parameters were accurately described. Vascular status was based on narrowing of the vessels, encasement, proximity of tumour, and circumference of tumour. Lymph nodes were assessed based on nodal shape, signs of necrosis, and short-axis diameter (<1 cm). In some cases direct cholangiography, by either PTC or ERCP, was also used to assess the extent of proximal ductal involvement.

We then evaluated the prognostic impact of the different variables on the main outcome parameters, namely, resectability rate, yield of laparoscopy, and incidence of metastasis. 

### 2.4. Criteria of Resectability

During the study period, we used the same criteria for resectability as described by Jarnagin et al. [19]. However, we do not consider bilateral segmental ductal involvement any longer a criterion of unresectability, nor tumor invasion in the bifurcation of the portal vein [20]. Hence, we applied the following criteria for resectability: absence of hepatic or extrahepatic metastases, with the exception of lymph nodes metastasis confined to the hepatoduodenal ligament. In addition, a future remnant liver featuring sufficient volume (30–40%) is required, in combination with tumor-free portal venous, arterial supply, and options for biliary reconstruction. Reasons for unresectability of the patients were described earlier in more detail [13, 18]. Patients were ultimately scored as resectable, when R0 or R1 resection had been performed.

### 2.5. Survival

Survival data was obtained from our own database or that of local hospitals and updated if necessary, by contacting the primary care physicians. Furthermore, additional survival data were collected through contacting registry databases. Survival (in months) was measured from the date of initial presentation at our center to the date of death, or the date of last contact when alive. 

### 2.6. Statistical Analysis

The data were analyzed with the SPSS version 16.0 software (SPSS Inc., Chicago, IL). 

We used univariate logistic regression to calculate odds ratios of portal vein involvement, hepatic artery involvement, lobar atrophy, suspicious lymph nodes, and bilateral ductal involvement for resectability, yield of laparoscopy, and presence of metastasis. Survival probabilities were estimated using the Kaplan-Meier method and compared by the logrank test. *P* values of <0.05 were considered as statistically significant.

## 3. Results

### 3.1. Patients

**Figure 1:**
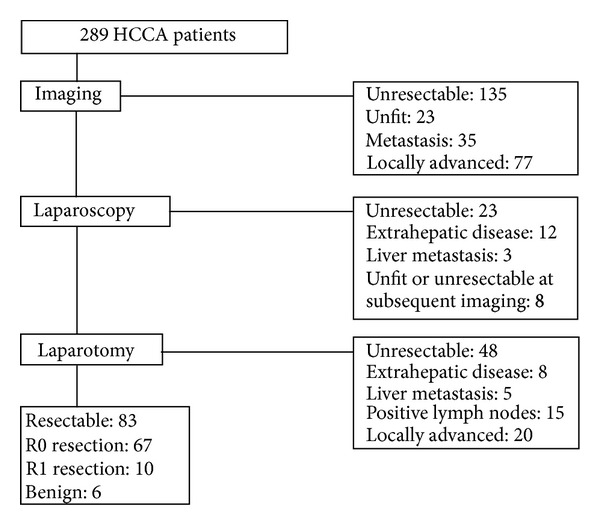
Patients with HCCA evaluated in the Academic Medical Center from January 2003 through August 2010.

As shown in Figure 1, 289 HCCA patients were seen in our center from January 2003 through August 2010. There were 183 men (63%) and 106 women (37%). The median age was 66 years (range 33–91). Preoperatively, patients were classified by the Bismuth-Corlette classification as type I (*n* = 19), type II (*n* = 20), type IIIa (*n* = 100), type IIIb (*n* = 55), or type IV (*n* = 95). 135 (47%) patients were considered unresectable (according to the resectability criteria described earlier) based on imaging findings that were usually confirmed by cytological puncture. Also 23 patients were unfit for major surgery, predominantly because of age and significant comorbidities. Another 23 patients were considered unresectable at staging laparoscopy, or by subsequent imaging after staging laparoscopy. 131 (45%) patients underwent explorative laparotomy, of whom a resection could be performed in 83 (63%) patients. The 48 patients who were deemed unresectable during laparotomy had metastases (*n* = 13), positive lymph nodes outside the hepatoduodenal ligament (*n* = 15), or locally advanced tumor (*n* = 20). The sites of positive lymph nodes precluding resection were celiac trunk in 5 patients, peripancreatic in 3 patients, periduodenal in 1 patient, and common hepatic artery in 6 patients (not resectable by regional lymphadenectomy). Resection encompassed hilar resection in combination with (extended) hemihepatectomy including segment 1 in 71 (86%) patients or hilar resection only in 12 (14%) patients. 32 patients underwent (extended) left hemihepatectomy, 36 underwent (extended) right hemihepatectomy, and 3 patients underwent a central liver resection.

### 3.2. Preoperative Parameters

The prognostic impact of the preoperative parameters is shown in Table 1. The presence of suspicious lymph nodes seemed most important for prediction of resectability. Bilateral ductal involvement (BC type IV) seemed important for the presence of metastasis, and consequently for the yield of laparoscopy. The involvement of the portal vein often coincided with involvement of the hepatic artery, yet the involvement of the hepatic artery seemed more predictive of unresectability as well as for incidence of metastasis.

### 3.3. Staging Systems

All potentially resectable patients based on imaging were staged according to the BC classification, and the MSKCC staging system. Results are shown in Table 2. Both systems show a significant correlation of stage and resectability. The BC classification also correlated with the incidence of metastasis, with an incidence of 40% in the type IV patients as compared to 12% in the type I and II patients. The yield of laparoscopy and overall survival was not statistically different between the staging groups. In both groups the high stage patients (T3, and BC type IV) represented only a small proportion of the resectable patients (16%).

### 3.4. Survival

**Figure 2:**
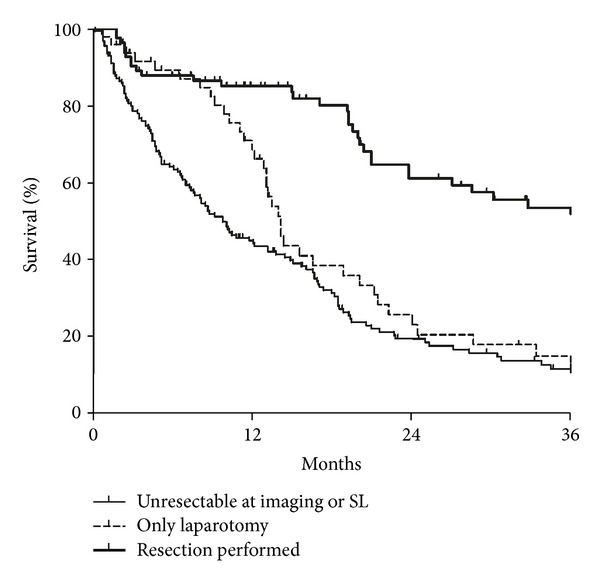
Survival of resected HCCA patients and patients who were unresectable at imaging, or laparoscopy or at laparotomy. Survival was significantly better in resectable patients (*P* < 0.001).

Complete survival data could be retrieved from all but 2 patients. At the time of analysis, 91 patients were still alive. Median survival for all patients (including unresectable patients) was 16 months. Patients with benign disease at final pathology (*n* = 6) were not included in this assessment. As expected, patients who had undergone resection had a significantly longer median survival than those who did not undergo resection (37 versus 14 months; *P* < 0.001), as shown in Figure 2. As a result, survival differences found within the different groups of the staging systems will very likely be a result of differences in resectability. Nonetheless, neither the MSKCC staging system nor the BC type did correlate with overall survival, as shown in Table 2.

## 4. Discussion

This study represents the common denominator of 289 patients referred to our center with HCCA during a 7-year period. The large number of patients enabled us to evaluate different preoperative factors important for resectability, metastasis, and yield of laparoscopy and to evaluate the current staging systems. 

Since the recognition of the disease in 1965 by Gerald Klatskin, much progress in management has been made, including developments in imaging, surgical techniques, and pre- and postoperative care. These developments have markedly increased the 5-year survival rate of patients who underwent resection to over 40% in recent series, including our own. Also, as mentioned above, two different classification systems have been introduced in the past, that is, the BC classification system and the MSKCC preoperative staging system [2, 3, 7], offering the possibility to compare data between centers, stratify patients before surgery in subgroups with different resectability rates, and select patients at risk who could benefit from additional imaging or staging laparoscopy [21]. Considering the latter, the additional value of staging laparoscopy has decreased dramatically in the last decade with improvements in imaging [13]. The BC classification system is based on proximal ductal infiltration of the tumor and is still very useful in communications within a hospital as well as between centers. Yet, its correlation with resectability or survival has been questioned, because it is more a classification system than a staging system [7]. In 1997 the MSKCC introduced a preoperative staging system, which also incorporated involvement of the portal vein and the presence of lobar atrophy. This group also pioneered the definition of resectability criteria, which differed largely between centers. Furthermore, they showed that their proposed staging system could predict resectability, need for hepatic resection, and survival. Yet, this system also has its limitations: the system is complicated and does not include in its assessment the presence of nodal or distant metastases or the involvement of the right or left hepatic artery [22]. However most importantly, patients are divided into three categories that also include patients that are unresectable based on imaging. Since the system was designed with the aim of predicting resectability, the inclusion of unresectable patients in the staging groups is less appropriate. We have also staged our patients according to the MSKCC classification, as shown in Table 2. The system predicted resectability, yet the predictive power for the other parameters (metastasis, yield of laparoscopy, and survival) was less impressive and not significant. The MSKCC system incorporated lobar atrophy and portal vein involvement in addition to proximal ductal infiltration as classified in the BC system, yet according to our results, involvement of the right or left hepatic artery was more predictive than portal vein involvement. Therefore, the incorporation of invasion of the right or left hepatic artery should probably be added to the system. Furthermore, the presence of suspicious lymph nodes obviously also correlated with resectability and should probably also be used to predict resectability. Lobar atrophy, however, was not predictive of one of the categories and therefore seems a less essential component of the system.

 In addition to the MSKCC and BC classification systems, a new staging system was recently proposed by Deoliveira et al. [22]. This system is designed to consider the extent of cancer in the bile duct, portal vein, and hepatic artery, as well as tumor size, nodal involvement, future remnant liver size, and distant metastases, as well as the volume of the putative remnant liver after resection. The authors herewith aim to standardize the reporting of HCCA and, consequently, allow comparisons of studies among centers or over time. In addition, the authors opened a registry, in which centers can prospectively and in a standardized fashion record features of this rare disease. Since the system is merely designed to standardize reporting, rather than predicting resectability and guiding therapy, the system is more a classification system than a staging system. 

 We analyzed 289 consecutive patients with HCCA presenting in our centre during a 7-year period until 2010. This allowed us to secure to some extent a homogeneity in patient evaluation, surgical treatment, and imaging protocols. We aimed to include all preoperative data, of which we expected any influence on resectability, on yield of staging laparoscopy or on presence of metastasis, and to evaluate their individual impact. However, there are two important limitations of this approach. Firstly, these preoperative parameters have their own accuracy, or inaccuracy. Imaging has much improved in the last years, and all patients are usually evaluated by Doppler ultrasound as well as state-of-the-art cross-sectional imaging. Yet, accuracy is still not 100% for these imaging tools, and consequently, the accuracy of the analysis will suffer from the inaccuracy of the imaging modalities used. Particularly the assessment of nodal status is challenging, as we previously evaluated [23]. Yet, this limitation applies to all preoperative parameters and, unfortunately, is hardly avoidable. In the future, well-performed imaging studies should assess sensitivity, specificity, and accuracy of the different imaging modalities. Until now, evidence on the accuracy of imaging studies for staging of HCCA is scarce and inconclusive [24]. Secondly, resection criteria are not universally adopted worldwide, and consequently, the primary outcome parameter in our study, that is, resectability, depends on the criteria used in our center. 

In conclusion, current staging systems do predict resectability, yet there is clearly room for improvement in patient selection. Future improved staging systems will hopefully enable us to select patients for additional treatment (adjuvant or neoadjuvant) or additional imaging or staging laparoscopy. According to our results, hepatic artery involvement and nodal status are also important for prediction of resectability, metastasis, and yield of laparoscopy and should therefore be incorporated in future staging systems after appropriate validation.

## Figures and Tables

**Table 1:** Preoperative parameters in HCCA patients predictive of resectability, metastases, and yield of laparoscopy.

		*n*	Unresectable rate (%)	OR (95% CI)	Yield of laparoscopy (%)	OR (95% CI)	Metastasis (%)	OR (95% CI)
Portal vein involvement	Yes No	68 86	63 47	2.0 (1.0–3.8)	9 15	0.7 (0.2–0.9)	16 22	0.7 (0.3–1.5)
Hepatic artery involvement	Yes No	78 76	65 42	2.6 (1.4–5.0)	10 13	0.7 (0.2–2.1)	21 17	1.3 (0.6–3.0)
Suspicious lymph nodes	Yes No	63 91	66 37	3.4 (1.7–6.6)	12 11	1.2 (0.4–3.4)	25 14	2.0 (0.9–4.6)
Bilateral involvement (BC type IV)	Yes No	26 128	57 39	2.1 (0.9–5.0)	20 9	2.5 (0.8–8.0)	40 15	3.6 (1.4–9.0)
Lobar atrophy	Yes No	20 134	55 50	1.2 (0.5–3.1)	11 11	0.9 (0.2–4.5)	15 19	0.7 (0.2–2.7)

**Table 2:** Patients staged according to the Bismuth-Corlette classification and MSKCC staging system.

Stage	Patients	Resected (%)	Metastasis (%)	Yield of laparoscopy (%)	Median survival (months)
MSKCC staging system					
T1	55	42 (71)	10 (17)	5/41 (12%)	21
T2	68	31 (45)	9 (13)	5/63 (8%)	19
T3	25	9 (36)	10 (40)	5/24 (21%)	23
*P* value		<0.01*	0.09*	0.43*	0.15**

Bismuth-Corlette classification					
Type I and II	26	18 (69)	3 (12)	1/12 (8%)	19
Type III	79	50 (52)	16 (17)	9/92 (10%)	17
Type IV	25	9 (36)	10 (40)	5/24 (21%)	12
*P* value		0.02*	0.01*	0.17*	0.12**

Patients with benign disease and patients with unresectable disease at imaging were not included in this assessment. *X ^2^ for trend; **logrank test.
